# Noise, air pollution exposure and attention-deficit/hyperactivity disorder: a meta-analysis

**DOI:** 10.3389/fpsyt.2026.1788310

**Published:** 2026-05-05

**Authors:** Jing Zhang, Xiaomeng Li, Yang Li, Yuefei Wu, Zitong Zhao, Yuxin Gao, Jian Zeng, Weijie Wang, Xiaolin Zhang

**Affiliations:** 1 Department of Medical Records, The Second Hospital of Hebei Medical University, Shijiazhuang, China; 2 Department of Epidemiology and Statistics, School of Public Health, Hebei Medical University, Hebei Province Key Laboratory of Environment and Human Health, Shijiazhuang, China; 3 Department of Neurosurgery, Hebei General Hospital, Shijiazhuang, China

**Keywords:** ADHD, air pollution, attention-deficit/hyperactivity disorder, meta-analysis, noise

## Abstract

**Objective:**

This meta-analysis evaluated the associations between noise exposure, air pollutants, and attention-deficit/hyperactivity disorder (ADHD) in children, aiming to inform future prevention strategies.

**Methods:**

Studies were systematically retrieved from CNKI, Wanfang, PubMed, Web of Science, Embase, and the Cochrane Library, covering publications from inception to November 2025. Heterogeneity was assessed using Cochran’s Q test and the *I²* statistic. Subgroup analyses, meta-regression, and sensitivity analyses were performed to evaluate the robustness of the findings.

**Results:**

Noise exposure was associated with a small increase in ADHD risk (odds ratio [*OR*] = 1.03, 95% confidence interval [CI]: 1.01–1.05), with stronger associations for childhood exposure, whereas prenatal exposure showed no significant effect. Given the modest effect size, this finding should be interpreted cautiously. Particulate matter (PM_2.5_ and PM_10_) was significantly associated with ADHD in continuous-exposure models—PM_2.5_ (*OR* = 1.32, *95% CI:* 1.16–1.50) and PM_10_ (*OR* = 1.47, *95% CI:* 1.15–1.87). In dichotomous models, PM_2.5_ was not significant, while PM_10_ remained positively associated (*OR* = 1.58, *95% CI:* 1.11–2.26). Elevated nitrogen dioxide (NO_2_) exposure was also associated with a modest increase in ADHD risk (*OR* = 1.11, *95% CI:* 1.02–1.20), whereas nitrogen oxides (NO_x_), ozone (O_3_), and sulfur dioxide (SO_2_) did not show significant associations.

**Conclusions:**

Noise and several air pollutants (PM_2.5_, PM_10_, and NO_2_) were significantly associated with increased ADHD risk, particularly during childhood exposure. Other pollutants, including O_3_ and SO_2_, did not demonstrate significant effects. These findings suggest that environmental noise and several air pollutants may be associated with ADHD; however, some observed associations, particularly for noise and NO_2_, were modest in magnitude and should be interpreted cautiously. These results reflect observational associations rather than evidence of a strong or causal effect, while the evidence for some pollutants remains limited or inconclusive. Further research is needed to clarify pollutant-specific associations and the role of exposure timing.

**Systematic Review Registration:**

https://www.crd.york.ac.uk/PROSPERO/view/CRD42024593274, identifier CRD42024593274; https://www.crd.york.ac.uk/PROSPERO/view/CRD42025632899, identifier CRD42025632899.

## 1 Introduction

Attention-Deficit/Hyperactivity Disorder (ADHD) is a common neurodevelopmental condition characterized by enduring deficits in attentional control, heightened impulsivity, and pronounced hyperactive behaviors (1, 2). Its global burden has increased markedly in recent years. A meta-regression published in 2007 estimated a worldwide prevalence of 5.29% (3), while a more recent synthesis of 61 cross-sectional studies reported prevalence rates of 7.6% in preschool and school-age children and 5.6% among adolescents (4). Although genetic susceptibility plays an important role in the onset of ADHD, growing research has also highlighted the potential relevance of environmental exposures (5–7).

Rapid urban expansion has intensified multiple environmental stressors, including environmental noise and air pollution (8, 9). Long-term exposure to environmental noise has been associated with externalizing behaviors in children, particularly hyperactivity (10–12). Exposure to air pollutants during pregnancy or early life may also interfere with neurodevelopment and has been linked to cognitive and behavioral difficulties (13, 14). Because noise and air pollution frequently co-occur—especially in urban traffic environments—children are often simultaneously exposed to multiple environmental stressors that may affect attention regulation, emotional control, and behavioral development.

Although the biological mechanisms linking environmental exposures to ADHD are not yet fully understood, several hypotheses have been proposed. Environmental noise may contribute to chronic stress responses, sleep disturbance, and impaired cognitive restoration, whereas air pollutants may affect neurodevelopment through pathways involving oxidative stress, systemic and neuroinflammation, disruption of the blood-brain barrier, and altered neuronal signaling. These mechanisms remain under investigation, but they provide biologically plausible pathways through which environmental exposures may be related to ADHD-related outcomes.

Despite growing interest in environmental determinants of ADHD, the existing review literature remains fragmented. Previous systematic reviews have often focused on a single exposure domain, particularly air pollution, specific pollutants such as PM_2.5_, or a limited developmental period such as pregnancy or early childhood. Relatively few evidence syntheses have considered environmental noise and air pollution within the same framework, even though these exposures frequently co-occur in urban settings. As a result, it remains unclear whether the strength of evidence is comparable across exposure types, whether associations differ by exposure window, and how consistent the findings are across geographic settings.

Against this background, the present meta-analysis extends earlier evidence in three main ways. First, it synthesizes findings for both environmental noise and multiple major air pollutants in relation to ADHD, allowing a broader comparison across exposure domains. Second, it explicitly examines temporal exposure windows, particularly prenatal versus childhood exposure, to assess whether developmental timing may modify the observed associations. Third, it draws on studies from a broad range of countries and regions, thereby providing a more geographically comprehensive summary of the available evidence. Although environmental noise and air pollution frequently co-occur, the present meta-analysis did not evaluate their joint or interactive effects directly. Instead, noise and air pollutants were examined separately within a single analytical framework, allowing us to identify where the evidence appears relatively consistent and where it remains limited or inconclusive.

Because ADHD was assessed differently across the included studies—most commonly through clinical diagnosis or standardized rating scales—variation in outcome ascertainment was considered an important methodological issue in the present review. Accordingly, this study aimed to systematically evaluate the associations between objectively measured environmental noise and major air pollutants and ADHD in children and adolescents. Specifically, we examined whether these associations differed across exposure types, developmental exposure windows, study characteristics, and methods of ADHD outcome assessment.

## 2 Methods

### 2.1 Literature search strategy

This study synthesized evidence from two pre-registered systematic reviews and meta-analyses. The noise-related review was registered in PROSPERO (CRD42024593274) https://www.crd.york.ac.uk/PROSPERO/view/CRD42024593274, and the air pollution review was registered in PROSPERO (CRD42025632899) https://www.crd.york.ac.uk/PROSPERO/view/CRD42025632899. A comprehensive literature search was conducted in the China National Knowledge Infrastructure (CNKI), Wanfang Database, PubMed, Embase, Web of Science, and the Cochrane Library from database inception to November 2025. Search strategies combined subject headings and free-text terms related to environmental noise, air pollution, and ADHD.

No formal language restriction was applied during the initial database search; however, only studies with sufficient information available in Chinese or English for screening and data extraction were included in the final review. In addition, the reference lists of relevant reviews and eligible articles were screened to identify potentially missed studies.

Screening was performed in two stages. First, titles and abstracts were reviewed to exclude clearly irrelevant records. Potentially eligible studies then underwent full-text assessment based on study design, exposure assessment, and outcome definition. All retrieved records were managed using EndNote X9.

### 2.2 Criteria for inclusion and exclusion

#### 2.2.1 Inclusion criteria

1. Studies involving children or adolescents younger than 18 years.
2. Objective environmental exposure assessment, including road traffic, aircraft, or multisource transportation noise measured by sound level meters or Geographic Information System (GIS) modeling, as well as quantitative monitoring data for major World Health Organization-recognized pollutants (e.g., NO_2_, PM_2.5_, and O_3_).
3. ADHD outcomes defined by clinical diagnosis based on DSM-IV or DSM-5 criteria and/or by standardized rating scales or questionnaires completed by parents, teachers, or clinicians.

#### 2.2.2 Exclusion criteria

1. Studies focusing on adults (≥18 years), experimental animal studies, or mixed-age samples for which child-specific data were unavailable.
2. Studies that treated noise or air pollution as subjective perception measures rather than objective environmental exposures, or intervention studies in which environmental exposure was included only as a control variable.
3. Studies reporting non-specific outcomes, such as physical impairments (e.g., reduced lung function) or psychiatric outcomes other than ADHD, without specific ADHD-related results.

### 2.3 Data extraction

A structured data extraction form was developed according to the study objectives. Two trained reviewers independently extracted the data and cross-checked the results. Disagreements were resolved through discussion or consultation with a third reviewer. For studies with missing key information, additional details were sought by contacting the corresponding authors or consulting clinical trial registries. Extracted variables included author name, publication year, participant characteristics, sample size, exposure metrics, ADHD diagnostic methods, types and sources of noise and pollutants, and main findings. ADHD outcome assessment methods were additionally classified as diagnosis-based or scale-based for use in subgroup analyses and meta-regression.

### 2.4 Quality assessment

Both cross-sectional and cohort studies were included in this review. Cross-sectional studies were appraised using the Agency for Healthcare Research and Quality (AHRQ) criteria, which comprise 11 methodological items scored as 1 if the criterion is met and 0 otherwise. Total scores were categorized as low (0–3), moderate (4–7), or high quality (8–11). Cohort studies were evaluated using the Newcastle–Ottawa Scale (NOS), which awards up to one star for each item and up to two stars for comparability, with a maximum of nine stars. A score of ≥7 was considered indicative of high methodological quality. Any disagreements between reviewers were resolved through discussion or consultation with a third evaluator.

### 2.5 Statistical analysis

Effect estimates extracted from the included studies comprised hazard ratios (HRs), odds ratios (ORs), and relative risks (RRs). To improve comparability across studies, these estimates were harmonized before pooling, with ORs treated as the primary summary measure. When RRs or HRs were reported, they were treated as approximate equivalents of ORs under the rare-outcome assumption and pooled on the logarithmic scale. This was considered reasonable because ADHD is a relatively uncommon outcome in most source populations and the reported effect sizes were generally modest.

Where baseline risk, cumulative incidence, or person-time information was unavailable, exact conversion between effect measures was not feasible. Therefore, published adjusted RRs and HRs were retained as approximate ORs after harmonizing exposure direction and scale. No further correction for person-time was applied because such information was not consistently reported across studies.

Heterogeneity was assessed using Cochran’s Q test (α = 0.10) and the *I²* statistic. A fixed-effect model was used when heterogeneity was low, whereas a random-effects model was applied when substantial heterogeneity was present. Subgroup analyses were conducted according to exposure window, study design, exposure assessment approach, and ADHD outcome assessment method (diagnosis-based vs. scale-based), where sufficient studies were available.

Meta-regression analyses were performed to explore potential sources of between-study heterogeneity. Study-level covariates considered in the analyses included study design, geographic region, publication year, exposure window, exposure assessment approach, and ADHD outcome assessment method, where sufficient data were available. Because the number of studies in several exposure-specific analyses was limited, meta-regression was conducted primarily as univariable analysis. Multivariable meta-regression was not performed because of limited statistical power and the potential for collinearity among study-level covariates. The main regression coefficients and corresponding *p*-values are reported in **Supplementary Tables S3** and **S4**.

Leave-one-out sensitivity analyses were performed to assess the robustness of the pooled estimates. Publication bias was evaluated by visual inspection of funnel plots and Egger’s test. All statistical analyses were performed using Stata version 15.1.

## 3 Results

Although several pooled associations reached statistical significance, some effect sizes were modest in magnitude and should be interpreted in light of both their methodological context and potential public health relevance.

### 3.1 Results of the literature search

**Figure 1:**
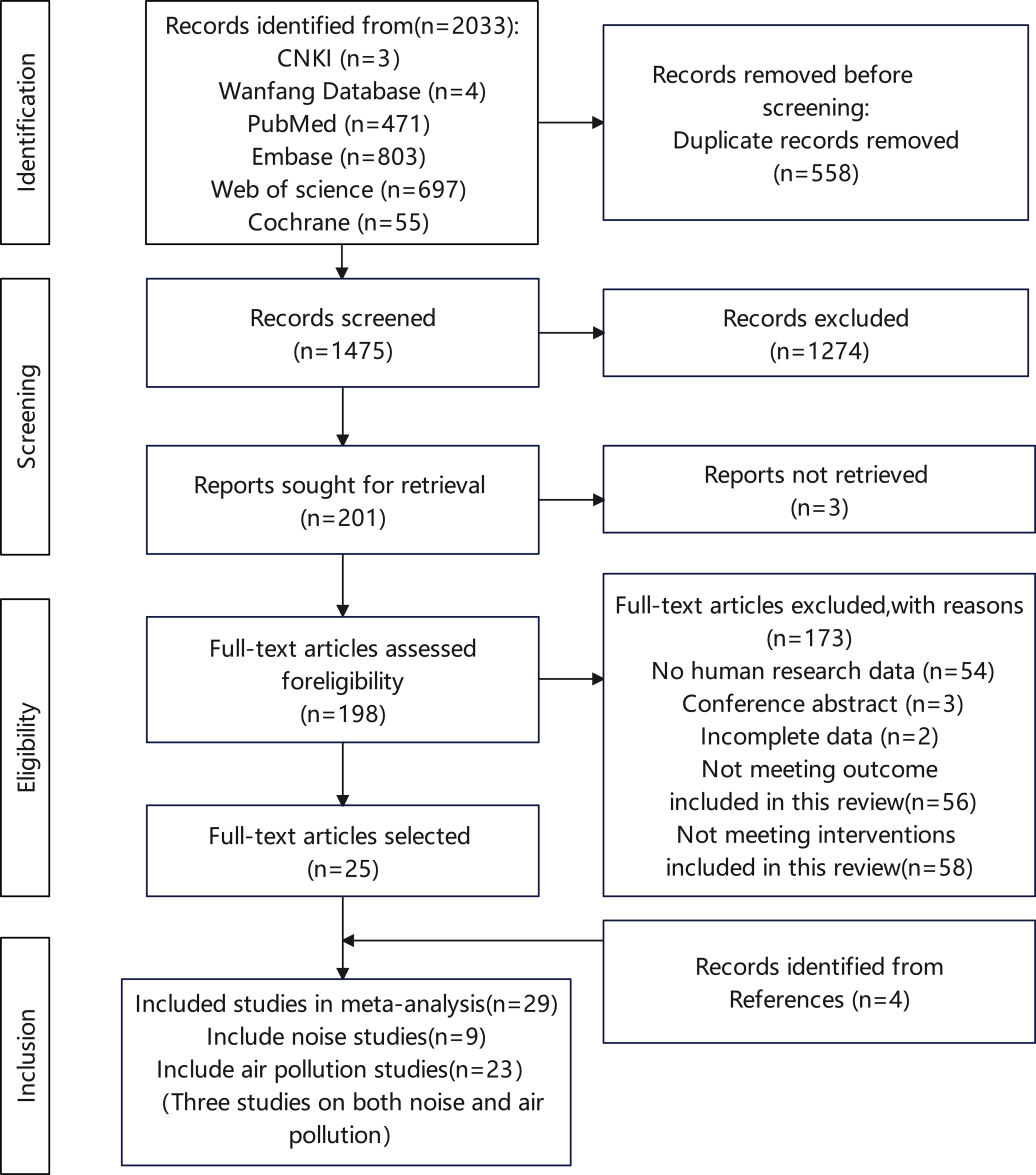
Literature search and screening process.

A comprehensive search identified records from CNKI (n=3), Wanfang (n=4), PubMed (n=471), Embase (n=803), Web of Science (n=697), and the Cochrane Library (n=55). After removing 558 duplicates, 1,475 unique records remained for screening. During the title and abstract review, 1,274 studies were excluded for failing to meet the PICOS framework, leaving 201 articles for full-text assessment. Of these, 176 were excluded for the following reasons: non-human research (n = 54), conference abstracts (n=3), insufficient data (n=5), absence of prespecified outcomes (n=56), or lack of relevant exposure measures (n= 58). Ultimately, 29 studies met the final inclusion criteria; among them, 25 were identified through database searches and 4 through reference screening. Nine studies involved noise exposure, 23 examined air pollution, and three assessed both exposures. Studies addressing both exposures were included in each respective analysis. The complete screening process and reasons for exclusion are displayed in the PRISMA flow diagram (**Figure 1**).

### 3.2 Basic characteristics of included studies

A total of 29 observational studies were included. Among these, 9 studies (31.0%) focused on noise exposure, 23 (79.3%) investigated air pollutants, and 3 (10.3%) assessed both environmental factors.

The noise-related studies comprised 102,638 participants, with the majority conducted in Europe (88.9%, 8/9). Study designs included prospective cohort studies (55.6%, 5/9) and cross-sectional analyses (44.4%, 4/9). For studies reporting multiple independent datasets, effect estimates adjusted for confounding variables were preferentially extracted to maximize comparability and methodological rigor (15).

The air pollution studies involved 1,779,758 participants from Asia (e.g., China, Korea), Europe (e.g., Spain, Germany), and North America (United States, Canada). Most were cohort designs (87.0%, 20/23), with the remainder being cross-sectional. Exposure assessment settings varied: 13.0% (3/23) monitored pollution in school settings, 87.0% (20/23) assessed residential exposure, and 4.3% (1/23) measured both home and school environments. The key features of included studies are summarized in **Table 1**.

**Table 1:** General characteristics of the included studies.

Author	Region	Year	Design	Sample size	Exposure type	Exposure assessment location	Diagnostic method
Zhou et al. (22)	China	2023	Cross-sectional study	35103	O3	School address	Scale-based
Choi et al. (23)	Korea	2023	Cohort study	329	SO2	Home address	Scale-based
Liu et al. (19)	China	2023	Cross-sectional study	164081	PM2.5	Home address	Scale-based
Li et al. (20)	Netherlands	2023	Cohort study	2481	Noise O3 SO2 NO2 PM2.5 PM10	Home address	Scale-based
Peterson et al. (24)	America	2022	Cohort study	332	PM2.5	Home address	Scale-based
Liu et al. (25)	China	2022	Cohort study	26052	SO_2_ NO_2_ O_3_ PM_2.5_ PM_10_	Home address	Scale-based
Chang et al. (26)	China	2022	Cohort study	425736	PM2.5	Home address	diagnosis
Fan et al. (18)	China	2022	Cohort study	98177	PM_2.5_ PM_10_	Home address	diagnosis
Yuchi et al. (27)	Canada	2022	Cohort study	28797	Noise PM2.5 NO2	Home address	diagnosis
Essers et al. (15)	Spain	2022	Cohort study	534	Noise	Home address	Scale-based
	Netherlands			7424	Noise		Scale-based
Maitre et al. (28)	England France Republic of Lithuania Spain Norway Greece	2021	Cohort study	1301	PM2.5	School address	Scale-based
Zijlema et al. (21)	Netherlands	2021	Cross-sectional study	2230	Noise	School address	diagnosis
							Scale-based
Thygesen et al. (29)	Denmark	2020	Cohort study	809654	PM2.5 NO2	Home address	diagnosis
Thygesen et al. (29)	Denmark	2020	Cohort study	809654	PM2.5 NO2	Home address	diagnosis
Shih et al. (30)	China	2020	Cohort study	16376	PM10 NOx SO2 NO NO2	Home address	diagnosis
Roberts et al. (31)	England Welsh	2019	Cohort study	284	PM2.5 NO2	Home address	Scale-based
Oudin et al. (32)	Southern	2019	Cohort study	48571	NOx	Home address	diagnosis
Markevychetal. (33)	Germany	2018	Cohort study	66823	PM10 NO2	Home address	diagnosis
Alemany et al. (34)	Europe	2018	Cohort study	2897	NO2	School address	Scale-based
Forns et al. (35)	Europe	2018	Cohort study	29127	PM10 NOX PM2.5 NO2	Home address	Scale-based
Min and Min (36).	Korea	2017	Cohort study	8936	NO2 PM10	Home address	diagnosis
Fuertes et al. (37)	Germany	2016	Cohort study	4745	NO2 PM10 PM2.5	Home address	Scale-based
Forns et al. (38)	Spain	2016	Cross-sectional study	2,897	Noise NO_2_	School address	Scale-based
Hjortebjerg et al. (39)	Denmark	2016	Cohort study	46940	Noise	Home address	Scale-based
Gong et al. (40)	Sweden	2014	Cohort study	3426	PM10 NOx	Home address	Scale-based
Tiesler et al. (41)	Germany	2013	Cohort study	872	Noise	Home address	Scale-based
Crombie et al. (42)	Europe	2011	Cross-sectional study	1900	Noise	School address	Scale-based
Siddique et al. (43)	India	2011	Cross-sectional study	1819	PM10	Home address	diagnosis
Morales et al. (44)	Spain	2009	Cohort study	398	NO2	Home address	Scale-based
Stansfeld et al. (45)	Europe	2009	Cross-sectional study	2844	Noise	Home address	Scale-based

### 3.3 Quality assessment of included studies

A total of 29 articles were assessed for quality using the AHRQ and the NOS for the evaluation of cross-sectional studies (7 articles) and cohort studies (22 articles). The specific scores are presented in **Supplementary Tables S1**, **S2**.

### 3.4 Association between noise exposure and ADHD

**Figure 2:**
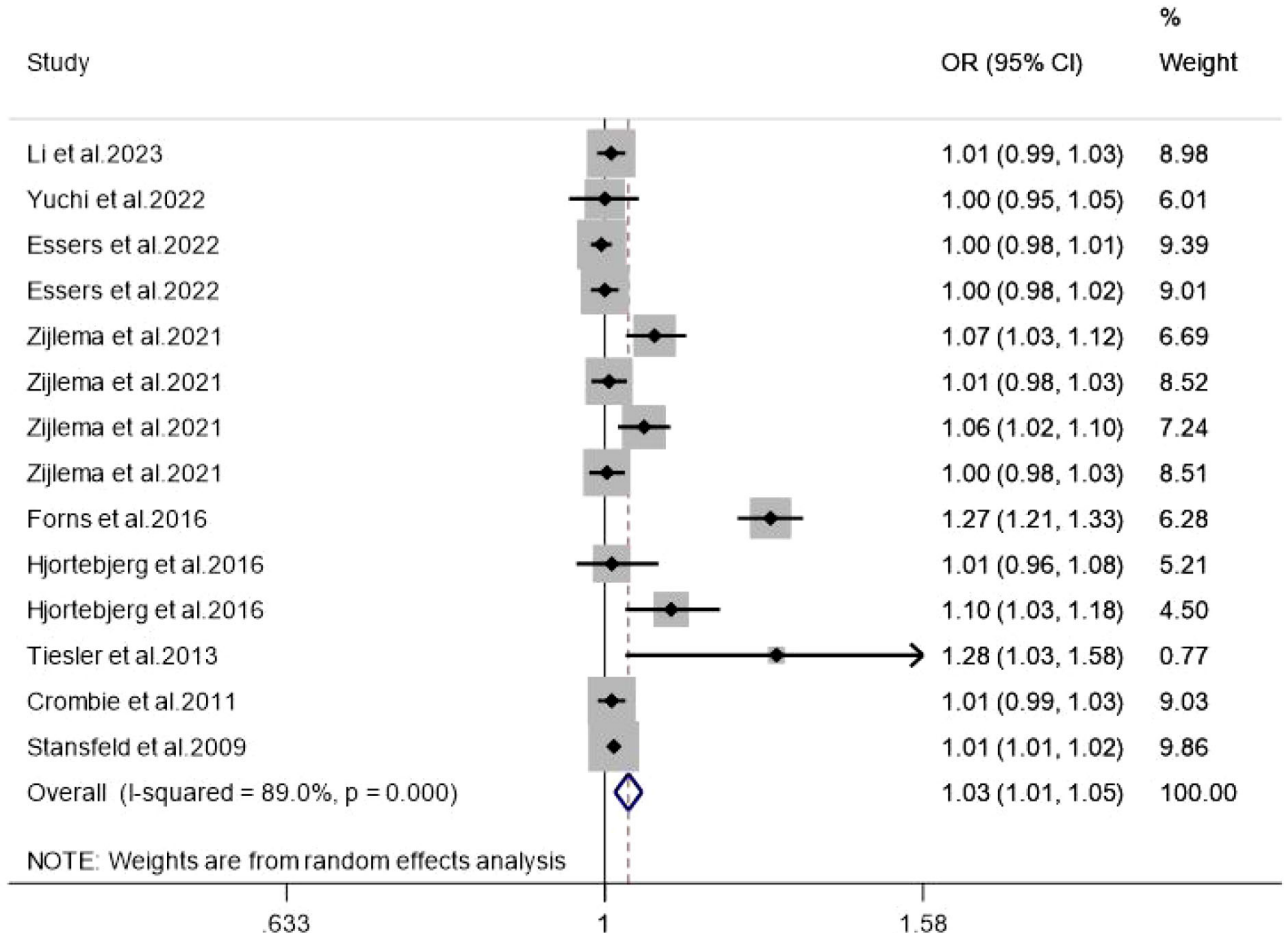
Association between noise exposure and ADHD.

This analysis ultimately included 14 independent effect estimates from 9 studies. The pooled analysis showed an overall OR of 1.03 (95% CI: 1.01–1.05) for the association between noise exposure and ADHD (**Figure 2**). Across the included studies, noise exposure was assessed using study-specific dB-based metrics (e.g., dB(A) or Lden), and some continuous estimates were modeled using study-specific exposure increments such as IQR increases; therefore, the pooled estimate is presented as an overall summary effect rather than per a single standardized increment. Although this pooled association was statistically significant, its magnitude was modest and should be interpreted in light of both its limited individual-level clinical impact and its potential population-level relevance. Given that the pooled *OR* was very close to the null value, this finding indicates only a weak association at the individual level and may partly reflect the influence of large cumulative sample sizes, between-study methodological differences, residual confounding, or chance.

Subgroup analyses were conducted according to exposure assessment location (Home address vs. School address), exposure window (Prenatal vs. Childhood), and diagnostic method (Scale-based vs. Diagnosis-based). Stronger pooled associations were observed for School address exposure assessment, childhood exposure, and diagnosis-based outcomes (**Table 2**). In particular, childhood exposure was positively associated with ADHD, whereas prenatal exposure was not.

**Table 2:** Subgroup analyses of the association between noise exposure and ADHD.

Subgroup	N	Pooled *OR (95% CI)*	*P for heterogeneity*	*I^2^* (%)
Exposure location				
Home address	9	1.02 (1.00-1.04)	0.004	65.0
School address	5	1.06 (1.01-1.11)	< 0.001	95.6
Exposure window				
Prenatal	2	1.00 (0.98-1.01)	0.630	0.00
Childhood	12	1.04 (1.02- 1.07)	< 0.001	90.1
Diagnostic method				
Scale-based	11	1.03 (1.01-1.05)	< 0.001	90.5
Diagnosis-based	3	1.05 (1.01-1.09)	0.089	58.8

Univariable meta-regression analyses for noise exposure are presented in **Supplementary Table S3**. Study design (*β* = 0.0175, *P* = 0.012), exposure assessment location (*β* = 0.0132, *P* = 0.046), exposure window (*β* = -0.0214, *P* = 0.015), and diagnostic method (*β* = -0.0351, *P* = 0.008) were significantly associated with between-study heterogeneity. Noise type showed borderline significance (*β* = 0.0063, *P* = 0.052), whereas region and publication year were not statistically significant predictors. These findings suggest that heterogeneity in the noise analyses was partly related to methodological differences across studies, particularly study design, exposure characterization, exposure timing, and outcome assessment.

Funnel plot inspection revealed no substantial asymmetry (**Supplementary Figure S1**), and Egger’s test indicated no evidence of publication bias (*P* = 0.121). Sensitivity analysis showed that the pooled estimate remained stable after sequential omission of individual studies, supporting the robustness of the findings (**Supplementary Figure S2**).

### 3.5 Association between air pollution exposure and ADHD

This study evaluated the associations between air pollutant exposure and ADHD using both continuous and categorical approaches. Method A (continuous exposure model) was used to assess exposure-response patterns across pollutant concentrations according to the increments reported in the original studies or the harmonized increment applied in the pooled analysis (e.g., per 10 µg/m³ increase or per IQR increase, where appropriate). Method B (categorical exposure model) compared ADHD risk between higher- and lower-exposure groups based on the exposure categories defined in the original studies.

#### 3.5.1 NO_2_

**Figure 3:**
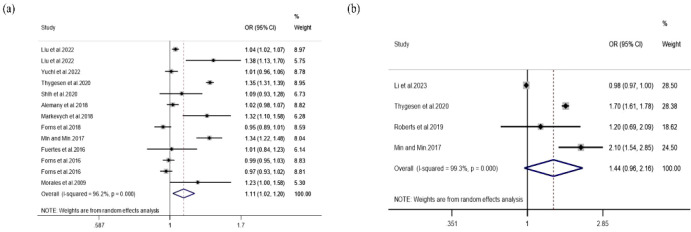
Association between NO_2_ exposure and ADHD.

A total of 17 independent effect estimates derived from 13 studies were included for NO_2_. Substantial heterogeneity was observed in both analytical approaches. Under the random-effects model, Method A yielded a pooled *OR* of 1.11 (95% CI: 1.02-1.20), whereas Method B yielded a pooled *OR* of 1.44 (95% CI: 0.96-2.16) (**Figure 3**). Although the Method A estimate reached statistical significance, the pooled *OR* remained close to 1.0, indicating that the magnitude of association was limited and should not be overstated.

Subgroup analyses suggested that heterogeneity varied according to exposure window and diagnostic method. Prenatal NO_2_ exposure was not significantly associated with ADHD (*OR* = 1.01, 95% *CI:* 0.94-1.09), whereas childhood exposure showed a modest positive association (*OR* = 1.12, *95% CI:* 1.00-1.26). Stratification by diagnostic method also showed divergent results, with diagnosis-based studies generally yielding stronger pooled estimates than scale-based studies (**Table 3**).

**Table 3:** Comparison of method A and method B for the association between NO_2_ and ADHD.

Subgroup	N	Pooled *OR (95% CI)*	*P for heterogeneity*	*I^2^* (%)
Method A	13	1.11 (1.02-1.20)	< 0.001	96.2
Exposure window				
Prenatal	3	1.01 (0.94-1.09)	0.022	73.9
Childhood	9	1.12 (1.00-1.26)	< 0.001	97.1
Prenatal and Childhood	1	1.38 (1.13-1.70)	–	–
Diagnostic method				
Scale-based	8	1.02 (0.97-1.06)	0.001	71.3
Diagnosis-based	5	1.21 (1.03-1.42)	< 0.001	96.2
Method B	4	1.44 (0.96-2.16)	< 0.001	99.3
Diagnostic method				
Scale-based	2	0.98 (0.97-1.00)	0.483	0.00
Diagnosis-based	2	1.79 (1.50-2.13)	0.184	43.3

Univariable meta-regression analyses for NO_2_ are presented in **Supplementary Table S4**. Among the examined covariates, diagnostic method was significantly associated with between-study heterogeneity (β=-0.1539, P = 0.022). Exposure assessment location showed a borderline association (β=-0.1455, P = 0.077), whereas region, publication year, study design, and exposure window were not statistically significant predictors. These findings suggest that differences in outcome ascertainment, and possibly exposure assessment location, may have contributed to heterogeneity in the NO_2_ analyses.

The funnel plot for Method A appeared generally symmetrical (**Supplementary Figure S3**), and Egger’s test indicated no significant publication bias (P = 0.964). Although the funnel plot for Method B appeared asymmetric (**Supplementary Figure S4**), Egger’s test did not suggest significant publication bias (P = 0.405). Sensitivity analyses showed that sequential exclusion of individual studies in both Method A and Method B did not materially alter the pooled estimates, supporting the robustness of the findings (**Supplementary Figure S5**).

#### 3.5.2 NO_x_

**Figure 4:**
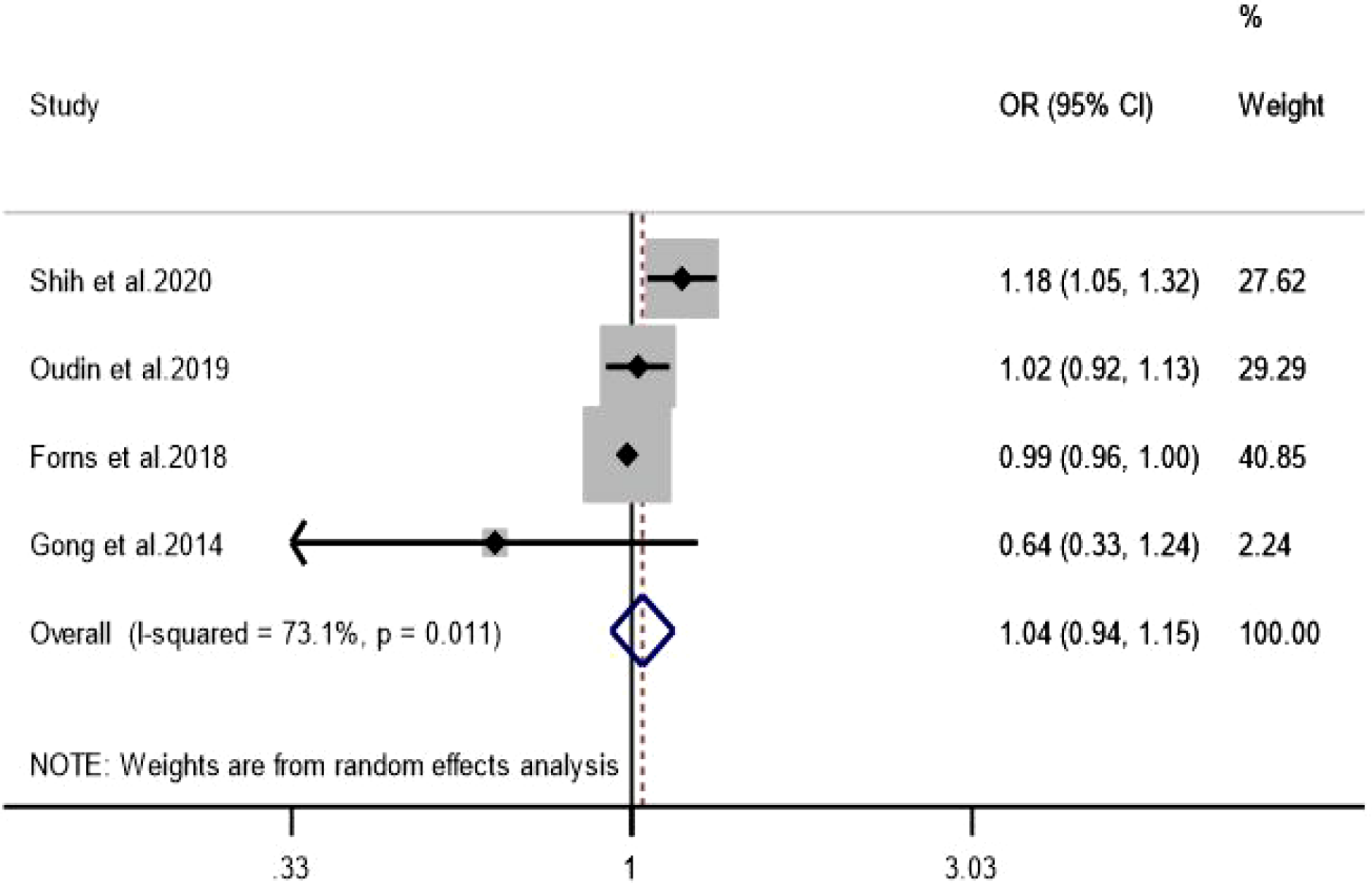
Forest plot of the association between NO_x_ exposure and ADHD.

Four independent effect estimates related to NO_x_ exposure were included in the analysis. The pooled analysis indicated no statistically significant association between NO_x_ exposure and ADHD (*OR* = 1.04, *95% CI:* 0.94-1.15) (**Figure 4**).

Subgroup analyses by diagnostic method suggested some variation in pooled estimates. Diagnosis-based studies yielded a pooled *OR* of 1.09 (*95% CI:* 0.95-1.26), whereas scale-based studies yielded a pooled *OR* of 0.91 (*95% CI:* 0.65-1.27) (**Table 4**). However, because only two effect estimates were available in each subgroup, these results should be interpreted cautiously.

**Table 4:** Subgroup analyses for the association between NO_x_ and ADHD.

Subgroup	N	Pooled *OR (95% CI)*	*P for heterogeneity*	*I^2^* (%)
Diagnostic method				
Scale-based	2	1.09 (0.95-1.26)	0.066	70.4
Diagnosis-based	2	0.91 (0.65-1.27)	0.011	38.6

The funnel plot appeared generally symmetrical (**Supplementary Figure S6**), and Egger’s test showed no evidence of publication bias (P = 0.689). Leave-one-out sensitivity analysis showed that sequential removal of individual studies did not materially alter the pooled estimate, supporting the robustness of the overall findings (**Supplementary Figure S7**).

#### 3.5.3 PM_10_

**Figure 5:**
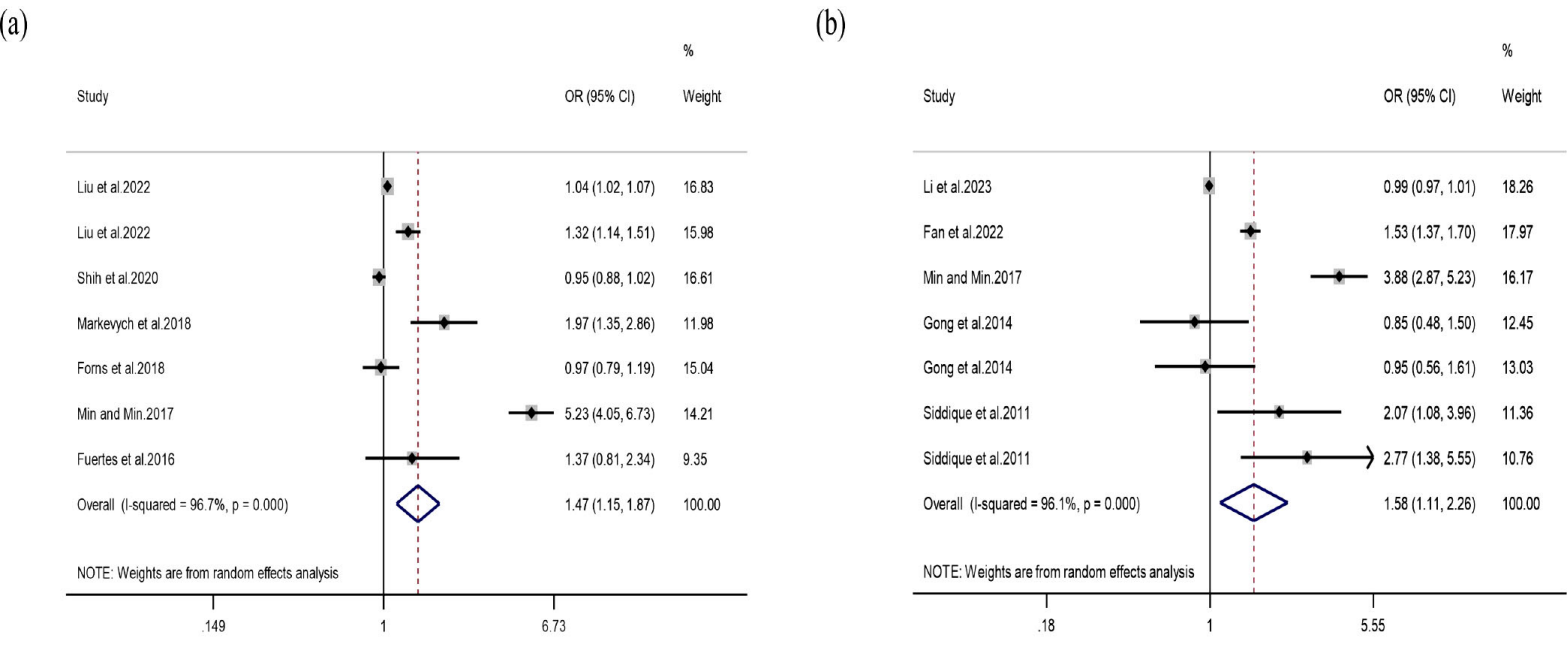
Association between PM_10_ exposure and ADHD. **(a)** Results obtained using Method A. **(b)** Results obtained using Method B.

Fourteen independent studies from ten articles were included for PM_10_. Method A yielded a pooled *OR* of 1.47 (95% CI:1.15-1.87). Method B produced a comparable effect estimate (*OR* = 1.58, 95% CI: 1.11-2.26), further supporting a potential relationship between higher PM_10_ concentrations and ADHD risk (**Figure 5**).

Subgroup analysis revealed that prenatal PM_10_ exposure was not significantly related to ADHD, whereas exposure during childhood showed a strong positive association (*OR* = 2.47, *95% CI:* 1.07-5.72). Studies using clinical diagnosis showed a stronger association (*OR* = 2.13, *95% CI:* 0.66-6.88). For Method B, the diagnostic subgroup showed a significant effect (*OR* = 2.39, *95% CI:* 1.33-4.31), suggesting inconsistency in diagnostic criteria (**Table 5**). Some individual studies reported markedly larger effect estimates than others, which likely contributed to the high heterogeneity observed in the PM_10_ analyses. However, leave-one-out sensitivity analysis did not materially alter the pooled estimates, suggesting that the overall findings were not driven by any single study.

**Table 5:** Subgroup analyses for the association between PM_10_ exposure and ADHD under method A and method B.

Subgroup	N	Pooled *OR (95% CI)*	*P for heterogeneity*	*I^2^* (%)
Method A	7	1.47 (1.15-1.87)	< 0.001	96.7
Exposure window				
Prenatal	3	1.00 (0.93-1.08)	0.054	65.7
Childhood	3	2.47 (1.07-5.72)	< 0.001	93.4
Prenatal and Childhood	1	1.32 (1.14-1.51)	–	–
Diagnostic method				
Scale-based	4	1.12 (0.96-1.31)	0.008	74.9
Diagnosis-based	3	2.13 (0.66-6.88)	< 0.001	98.8
Method B	7	1.58 (1.11-2.26)	< 0.001	96.1
Diagnostic method				
Scale-based	3	0.99 (0.97-1.01)	0.862	0.0
Diagnosis-based	4	2.39 (1.33-4.31)	< 0.001	91.4

The funnel plot for Method A and Method B exhibited slight asymmetry (**Supplementary Figures S8**, **S9**), although Egger’s test showed no significant publication bias (*P* = 0.196 and *P* = 0.104).

Sensitivity analysis demonstrated that sequentially omitting individual studies produced no meaningful changes in the pooled estimates for either method, confirming the robustness and stability of the random-effects models (**Supplementary Figure S10**).

#### 3.5.4 PM_2.5_

**Figure 6:**
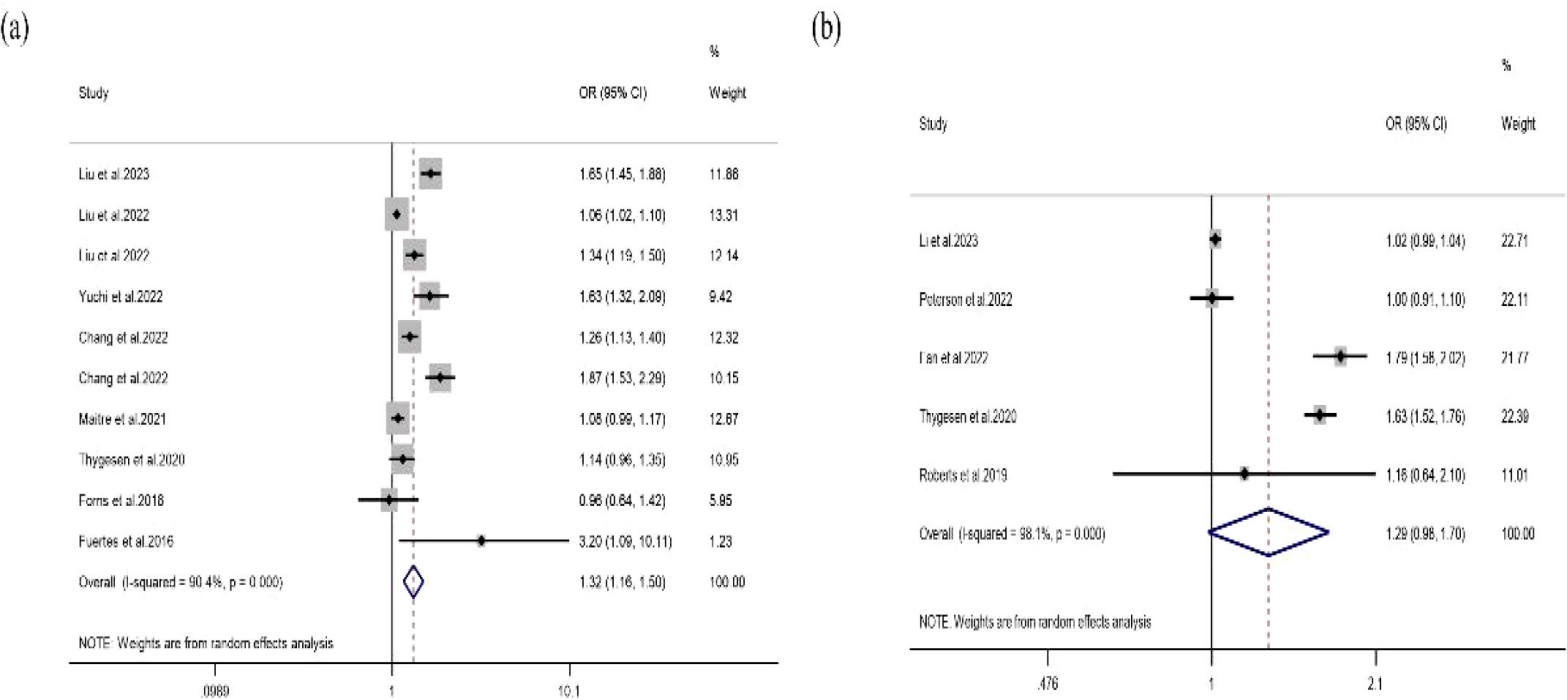
Association between PM_2.5_ exposure and ADHD. **(a)** Results obtained using Method A. **(b)** Results obtained using Method B.

Fifteen independent studies across twelve articles were included for PM_2.5_. The pooled *OR* from Method A was 1.32 (*95% CI:* 1.16-1.50, *P* < 0.05). For Method B, the pooled *OR* was 1.29 (*95% CI:* 0.98-1.70, *P* = 0.069) (**Figure 6**).

Subgroup analysis indicated a positive association for childhood exposure (*OR* = 1.47, *95% CI:* 1.17-1.86). Clinical diagnosis studies showed stronger associations (*OR* = 1.43, *95% CI:* 1.16-1.76), whereas rating scale studies also supported PM_2.5_ as a risk factor for ADHD (*OR* = 1.24, *95% CI:* 1.05-1.46). For Method B, diagnostic interview showed significant effects (*OR* = 1.69, *95% CI:* 1.54-1.84) (**Table 6**).

**Table 6:** Subgroup analyses for the association between PM_2.5_ exposure and ADHD under method A and method B.

Subgroup	N	Pooled *OR (95% CI)*	*P for heterogeneity*	*I^2^* (%)
Method A	10	1.32 (1.16-1.50)	< 0.001	90.4
Exposure window				
Prenatal	3	1.12 (0.97-1.30)	0.011	78.0
Childhood	6	1.47 (1.17-1.86)	< 0.001	90.2
Prenatal and Childhood	1	1.34 (1.19-1.50)	–	–
Diagnostic method				
Scale-based	6	1.24 (1.05-1.46)	< 0.001	91.0
Diagnosis-based	4	1.43 (1.16-1.76)	< 0.001	83.2
Method B	5	1.29 (0.98-1.70)	< 0.001	98.1
Diagnostic method				
Scale-based	3	1.02 (0.99-1.04)	0.871	0.00
Diagnosis-based	2	1.69 (1.54-1.84)	0.200	39.2

The funnel plot for Method A exhibited slight asymmetry, and Egger’s test (P = 0.026) indicated potential publication bias (**Supplementary Figure S11**). To address this, the trim-and-fill method was applied, and the adjusted pooled estimates changed only minimally (**Supplementary Figure S12**), supporting the robustness of the findings. For Method B (**Supplementary Figure S13**), the funnel plot suggested mild asymmetry due to a dispersed right tail; however, Egger’s test (*P* = 0.311) did not indicate significant publication bias.

Sensitivity analyses further confirmed stability: sequential exclusion of individual studies did not materially alter the pooled estimates for Method A or Method B (**Supplementary Figure S14**), demonstrating the reliability of both models.

#### 3.5.5 O_3_

**Figure 7:**
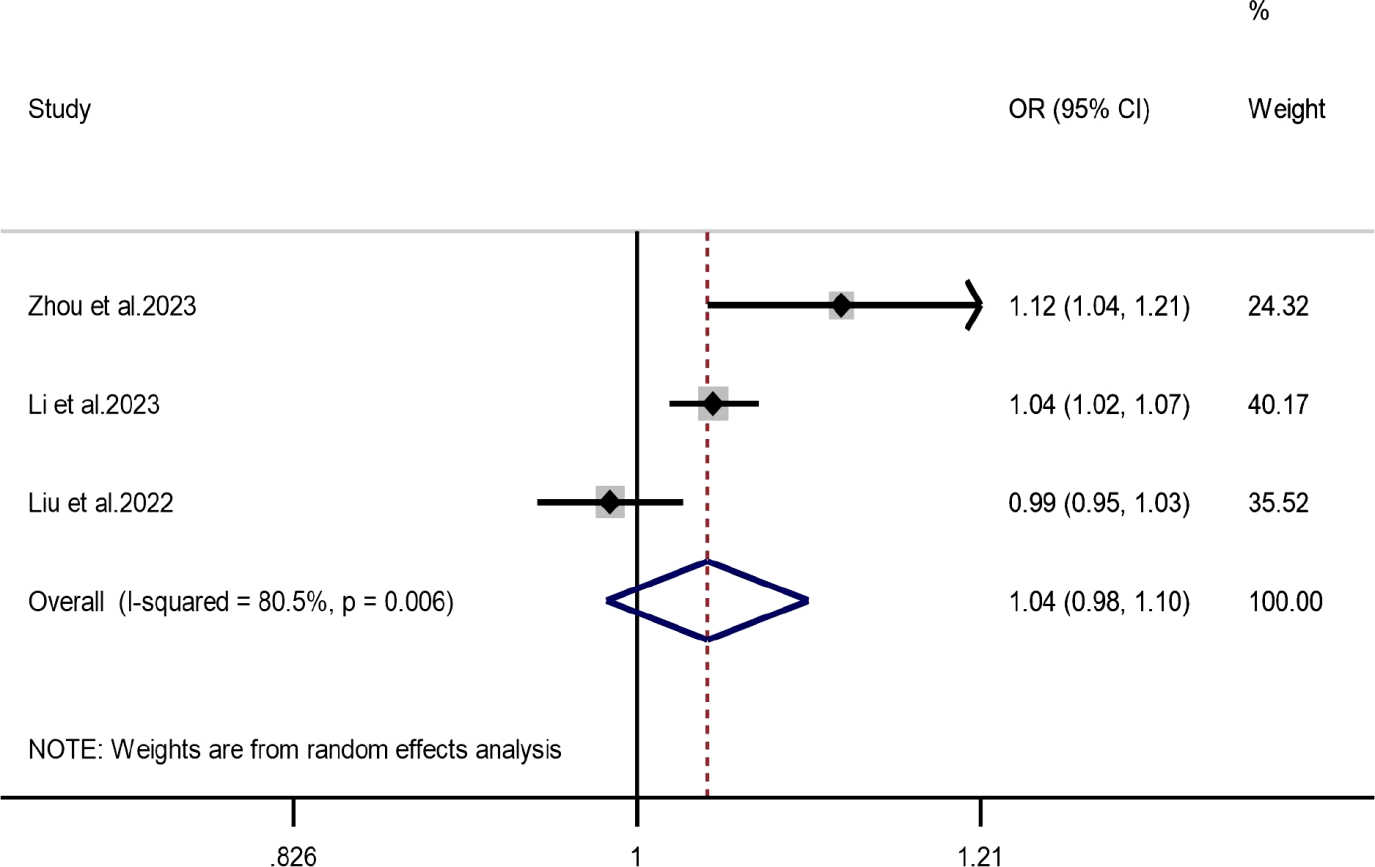
Association between O_3_ exposure and ADHD.

Three independent studies were included for O_3_. The pooled *OR* was 1.04 (*95% CI:*0.98–1.10), suggesting no statistically significant association between O_3_ exposure and ADHD (**Figure 7**). Because the number of included studies was limited, these results should be interpreted cautiously and are better regarded as inconclusive rather than definitively null. Funnel plot inspection and Egger’s test (*P* = 0.850) were of limited reliability in this context, while leave-one-out sensitivity analysis produced consistent estimates (**Supplementary Figures S15**, **S16**).

#### 3.5.6 SO_2_

**Figure 8:**
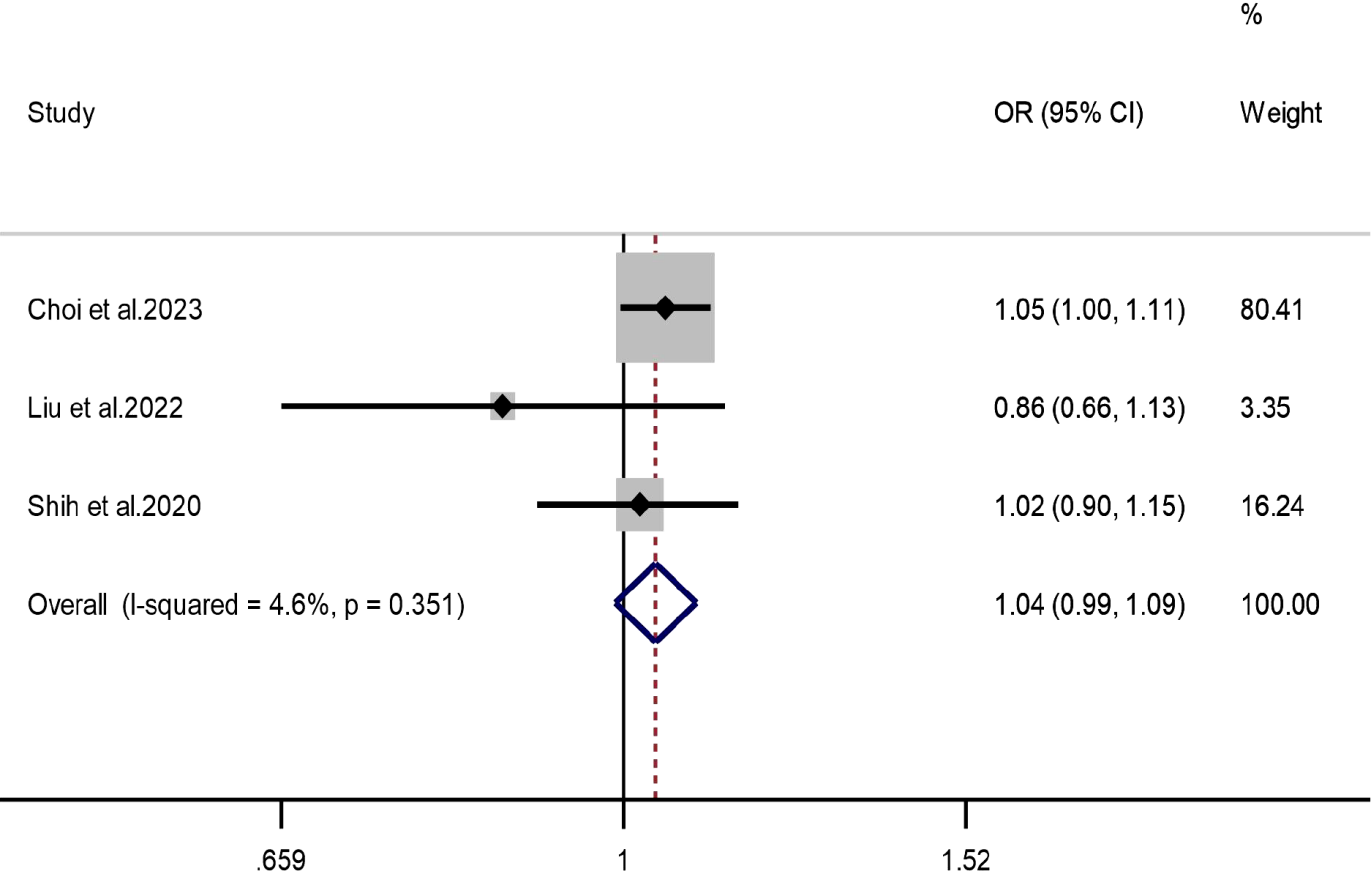
Forest plot of the association between SO_2_ exposure and ADHD.

Three independent studies were included for SO_2_, with minimal heterogeneity (*I²* = 4.6%, *P* = 0.351); therefore, a fixed-effect model was applied. The pooled *OR* was 1.04 (*95% CI:* 0.99–1.09), indicating no statistically significant association between SO_2_ exposure and ADHD (**Figure 8**). Because only a small number of studies were available, these findings should be interpreted cautiously and are better viewed as limited or inconclusive rather than as evidence of no effect. Funnel plot inspection and Egger’s test (*P* = 0.178) were also of limited interpretability in this setting, and leave-one-out sensitivity analysis produced consistent results (**Supplementary Figures S17**, **S18**).

### 3.6 Heterogeneity and subgroup analyses

Substantial heterogeneity was observed across several pooled analyses (**Supplementary Table S5**). Overall I² values were high for noise, NO_2_, PM_2.5_, and PM_10_, but lower for NO_x_. Subgroup analyses and meta-regression suggested that heterogeneity was partly related to exposure window, diagnostic method, study design, and exposure assessment approach. In general, stronger associations were observed for childhood exposure and for diagnosis-based outcomes. However, no single study-level characteristic fully explained the observed variability, indicating that the heterogeneity likely arose from multiple overlapping methodological and population-level factors.

Across the available subgroup and meta-regression analyses, diagnostic method and exposure window emerged as the most consistent contributors to between-study variability, whereas study design and exposure assessment approach appeared particularly relevant in the noise analyses.

## 4 Discussion

The present meta-analysis extends the existing literature on environmental determinants of ADHD by synthesizing evidence on both environmental noise and multiple major air pollutants within a single study framework. Previous reviews have often focused on selected pollutants, specific developmental periods, or broader neurodevelopmental outcomes rather than ADHD specifically. By contrast, our analysis allows a more direct comparison across exposure types and developmental windows, and provides a broader geographic summary of the available epidemiological evidence.

Overall, our findings suggest that the associations between environmental exposures and ADHD are not uniform across exposure domains or exposure periods. More consistent positive associations were observed for childhood noise exposure and for several air pollutants, particularly PM_2.5_, PM_10_, and NO_2_. In contrast, the evidence for prenatal noise exposure and for pollutants such as O_3_, SO_2_, and NO_x_ was more limited or inconsistent. Rather than demonstrating definitive causal effects, our findings support the interpretation that certain environmental exposures are associated with ADHD in children and adolescents, while uncertainty remains for several exposure categories. This distinction is important for interpreting the current evidence base and for guiding future studies toward more precise exposure assessment, better control of confounding, and closer examination of sensitive developmental windows (14, 16, 17).

Our findings further suggest that environmental pollution, particularly environmental noise and fine particulate air pollutants, is associated with ADHD in childhood. Noise exposure showed a consistent positive association with ADHD, with stronger associations observed for school-based exposure assessment and for childhood exposure. Higher concentrations of NO_2_, PM_10_, and PM_2.5_ were also associated with ADHD, particularly for childhood exposure. By contrast, the associations involving NO_x_, SO_2_, and O_3_ were weaker and remained limited or inconsistent. These findings should be interpreted cautiously and should not be taken as definitive evidence of no effect. Several factors may help explain the null or unstable results. First, the exposure range for these pollutants may have been relatively narrow in some study settings, limiting the ability to detect associations. Second, differences in co-pollutant adjustment across studies may have influenced the estimated effects, particularly because these pollutants are often correlated with other traffic- or combustion-related exposures. Third, substantial variation in the spatial and temporal resolution of exposure assessment models may have introduced additional exposure misclassification. In addition, the toxicological pathways most relevant to ADHD-related outcomes may differ across pollutants, and the available epidemiological evidence for O_3_, SO_2_, and NO_x_ remains comparatively limited. Because the number of studies for some of these pollutants was small, the corresponding pooled estimates are better regarded as exploratory or inconclusive rather than definitive. Therefore, the present findings are better interpreted as inconclusive rather than as evidence of no association (14, 16, 17).

A substantial body of research has suggested that noise exposure is associated with a range of behavioral disturbances in children, including inattention and impulsive behaviors. Findings from European and U.S. cohorts have suggested that traffic-related and environmental noise may be associated with poorer learning performance and reduced sustained attention in children. Children living in noisier environments have been reported to exhibit more ADHD-related symptoms or behavioral difficulties (4). In the current meta-analysis, a statistically significant association between noise exposure and ADHD was observed. The analysis also differentiated across exposure timing and environmental contexts, providing a more detailed view of how noise-related associations with ADHD may vary across settings. This approach provides a more refined perspective on the association between noise exposure and ADHD-related outcomes in childhood. At the same time, the practical importance of this finding should be interpreted cautiously. The observed association for noise exposure was statistically significant but modest in magnitude, and an effect size of this order should not be viewed as clinically decisive at the individual level. Importantly, effect estimates of this magnitude (e.g., *ORs* close to 1.0-1.1) indicate only weak associations. Such small relative effects may be sensitive to residual confounding, exposure misclassification, differences in study design and outcome ascertainment, and the influence of large cumulative sample sizes across pooled studies. Therefore, statistical significance in this context should not be interpreted as evidence of a strong or clinically decisive relationship. Accordingly, the main value of these findings lies in generating hypotheses and informing population-level environmental research, rather than supporting strong individual-level risk prediction or causal inference. Moreover, the substantial heterogeneity across studies indicates that the magnitude of this association varied considerably across methodological and population contexts. However, because environmental noise is a common and long-term exposure in many urban settings, even a small relative increase in risk may still be relevant from a public health perspective if large numbers of children are exposed.

In addition, existing literature has proposed that long-term exposure to airborne pollutants such as PM_2.5_ and NO_2_ may affect neurodevelopment through mechanisms including inflammatory responses and oxidative stress, which could be relevant to ADHD-related outcomes (18). Recent studies conducted in China, particularly in regions with severe air quality problems, have reported associations between elevated concentrations of PM_2.5_ and other pollutants and multiple adverse outcomes, including impaired cognition, emotional problems, and behavioral difficulties (19). Unlike studies that examine air pollution as a single exposure factor, this meta-analysis incorporated subgroup evaluations across multiple pollutants to clarify how exposure windows and diagnostic approaches may influence the observed associations with ADHD. Two analytic strategies were adopted: a continuous exposure model (Method A) to quantify exposure-response gradients and a categorical model (Method B) to compare associations between high- and low-exposure groups. Method A enabled a more nuanced interpretation of concentration-related risk gradients; however, the observational nature of the included studies precludes definitive causal inference. Method B provided a complementary perspective by highlighting potential exposure thresholds associated with differences in ADHD-related outcomes. The broadly consistent direction of associations across both approaches supports the robustness of the findings, while differences in effect magnitude may reflect variations in exposure categorization, between-study heterogeneity, or possible non-linear response patterns.

By integrating multiple analytic models and synthesizing data from diverse populations, geographic regions, and pollutant profiles, this study identified both consistent patterns and substantial variability in the relationship between environmental pollution and ADHD. To explore heterogeneity, the analysis incorporated subgroup assessments, meta-regression, and sensitivity testing, allowing a more nuanced interpretation of the pooled findings. The subgroup analyses further suggest that between-study heterogeneity was partly driven by differences in exposure window, diagnostic method, study design, and exposure assessment approach. These findings indicate that the pooled estimates should be interpreted cautiously when studies with different methodological characteristics are combined. Although the overall summary estimates remain informative, the subgroup patterns provide important context for understanding the variability across studies.

The findings of this review support associations between both noise and air pollution exposure and ADHD; however, several limitations related to study quality and methodological variability must be acknowledged. These findings should be interpreted as observational associations rather than evidence of direct causal effects. This distinction is particularly important given the modest magnitude of several pooled estimates, the limited number of studies for some exposures, and the substantial heterogeneity observed across analyses.

Considerable heterogeneity arose from differences in research design and exposure assessment approaches across studies. For instance, Li YR (20), Essers E (15), and Zijlema WL (21) all applied the equivalent continuous sound level (Lden) as a noise metric, yet their classification schemes differed substantially. Zijlema WL divided noise into three categories (<50 dBA, 50–60 dBA, >60 dBA), Essers E focused specifically on traffic noise, and Li YR incorporated a wider range of noise sources. The high heterogeneity observed for noise exposure likely stems from these inconsistencies in noise categorization. Similar discrepancies were present in assessments of NO_2_, PM_10_, and PM_2.5_ in Method B, where varying pollutant classifications may have affected the precision of exposure and response estimates. Thus, while the study’s conclusions are generally robust, the possibility of bias from individual studies cannot be entirely excluded. In addition, variation in methodological quality across studies may have influenced the interpretation of the pooled results. Studies with less precise exposure assessment, less standardized outcome ascertainment, or weaker control of confounding may have contributed disproportionately to uncertainty and heterogeneity, which should be considered when interpreting the overall findings. Additionally, since the majority of included research relied on observational designs, definitive causal inferences cannot yet be made.

Exposure quantification also posed challenges. In several studies, the reported pollutant concentrations did not fully align with the relevant exposure windows used for analysis. Seasonal fluctuations, meteorological conditions, and local environmental characteristics further complicate the estimation of true exposure levels. Moreover, incomplete information on children’s exact exposure locations and time-activity patterns may have reduced the accuracy of exposure assessment.

Residual confounding also remains an important concern. Although many included studies adjusted for major covariates, adjustment was not consistent across studies, and potentially important factors such as parental psychopathology, socioeconomic status, indoor environmental exposures, and co-exposures may not have been fully accounted for. In addition, both exposure and outcome misclassification are possible. Exposure estimates based on residential address, modeled concentrations, or area-level indicators may not fully reflect individual-level exposure, while ADHD ascertainment varied across studies and may have been influenced by differences in diagnostic methods, symptom scales, or healthcare access. Reverse causality and related selection processes also cannot be entirely excluded in observational studies, because family-level characteristics associated with child behavioral vulnerability may influence residential choice, environmental conditions, and the likelihood of diagnosis. These issues may have affected the magnitude of the observed associations and further support a cautious interpretation of the findings.

Most previous investigations have examined noise or air pollution independently, and only two studies addressed their combined effects. Yet in everyday environments, these exposures often occur simultaneously, raising the possibility of interactive or synergistic influences. Future research should therefore consider joint exposure models to better characterize how combined environmental stressors may relate to children’s neurodevelopment.

Another limitation is that different effect measures reported across studies (ORs, RRs, and HRs) had to be harmonized for pooling. In the absence of consistently reported baseline risk, cumulative incidence, and person-time data, some RRs and HRs were analyzed as approximate ORs under the rare-outcome assumption rather than being converted using exact formulas. Although this approach improved comparability across studies, it may also have introduced some approximation error.

Overall, this analysis provides quantitative evidence of associations between exposure to noise and air pollutants and ADHD, and offers an important foundation for subsequent research. Future investigations should prioritize clarifying whether these associations are likely to reflect causal relationships by using more rigorous designs, including well-characterized longitudinal cohort studies, improved exposure assessment, and more consistent control of confounding.

## 5 Conclusions

Noise exposure was associated with ADHD, with stronger associations observed for school-based exposure assessment and childhood exposure. NO_2_, PM_10_, and PM_2.5_ were also associated with ADHD, particularly during childhood exposure. However, the observed associations for noise and NO_2_ were modest in magnitude and should be interpreted cautiously, as they do not by themselves indicate a strong or causal relationship. In contrast, the findings for NO_x_, O_3_, and SO_2_ were weaker and should be interpreted as limited or inconclusive rather than definitively null.

## Data Availability

The original contributions presented in the study are included in the article/**Supplementary Material**. Further inquiries can be directed to the corresponding author.
