# Supplementary material for: Noise, air pollution exposure and attention-deficit/hyperactivity disorder: a meta-analysis
Source: Front Psychiatry. 2026 May 5;17:1788310. doi: 10.3389/fpsyt.2026.1788310 (PMC13185364; doi:10.3389/fpsyt.2026.1788310)
Supplement: Supplementary file 1 [file Table1.docx]

Table S1 The cross-sectional study included literature quality evaluation

| Author | Year | Quality grade | Total score |
| --- | --- | --- | --- |
| Liu et al. | 2023 | high quality | 8 |
| Zhou et al. | 2023 | high quality | 8 |
| Zijlema et al. | 2021 | high quality | 9 |
| Forns et al | 2016 | high quality | 8 |
| Crombie et al. | 2011 | moderate quality | 7 |
| Siddique et al. | 2011 | moderate quality | 7 |
| Stansfeld et al. | 2009 | moderate quality | 7 |

Table S2 The cohort study included literature quality evaluation

| Author | Year | Selection | Comparability | Outcome | Total score |
| --- | --- | --- | --- | --- | --- |
| Li et al. | 2023 | ★★★ | ★★ | ★★★ | 8 |
| Choi et al. | 2023 | ★★★ | ★★ | ★★ | 7 |
| Yuchi et al. | 2022 | ★★★★ | ★★ | ★★ | 8 |
| Essers et al. | 2022 | ★★★ | ★★ | ★★★ | 8 |
| Peterson et al. | 2022 | ★★★ | ★★ | ★★★ | 8 |
| Liu et al. | 2022 | ★★★ | ★★ | ★★★ | 8 |
| Chang et al. | 2022 | ★★★★ | ★★ | ★★★ | 9 |
| Fan et al. | 2022 | ★★★ | ★★ | ★★★ | 8 |
| Maitre et al. | 2021 | ★★★ | ★★ | ★★★ | 8 |
| Thygesen et al. | 2020 | ★★★★ | ★★ | ★★★ | 9 |
| Shih et al. | 2020 | ★★★★ | ★★ | ★★★ | 9 |
| Roberts et al. | 2019 | ★★★ | ★★ | ★★★ | 8 |
| Oudin et al. | 2019 | ★★★★ | ★★ | ★★★ | 9 |
| Alemany et al. | 2018 | ★★★ | ★★ | ★★ | 7 |
| Forns et al. | 2018 | ★★★ | ★★ | ★★★ | 8 |
| Markevych et al. | 2018 | ★★★ | ★★ | ★★★ | 8 |
| Min and Min. | 2017 | ★★★ | ★★ | ★★★ | 8 |
| Fuertes et al. | 2016 | ★★★ | ★★ | ★★★ | 8 |
| Hjortebjerg et al. | 2016 | ★★ | ★★ | ★★★ | 7 |
| Gong et al. | 2014 | ★★★ | ★★ | ★★★ | 8 |
| Tiesler et al. | 2013 | ★★ | ★★ | ★★★ | 7 |
| Morales et al. | 2009 | ★★★ | ★★ | ★★ | 7 |

Table S3. Univariable meta-regression analyses for the association between noise exposure and ADHD

| **Covariate** | **Regressioncoefficient** | **SE** | **Z** | ***P value*** |
| --- | --- | --- | --- | --- |
| Region | -0.0148 | 0.026 | -0.57 | 0.569 |
| Publication year | -0.0004 | 0.0005 | -0.81 | 0.417 |
| Noise type | 0.0063 | 0.0032 | 1.94 | 0.052 |
| Study design | 0.0175 | 0.0069 | 2.52 | 0.012 |
| Exposure assessment location | 0.0132 | 0.0066 | 2.00 | 0.046 |
| Exposure window | -0.0214 | 0.0088 | -2.43 | 0.015 |
| Diagnostic method | -0.0351 | 0.0131 | -2.67 | 0.008 |

Table S4. Univariable meta-regression analyses for the association between NO₂ exposure and ADHD

| **Covariate** | **Regressioncoefficient** | **SE** | **Z** | ***P value*** |
| --- | --- | --- | --- | --- |
| Region | -0.0041 | 0.0124 | -0.33 | 0.743 |
| Publication year | 0.0019 | 0.0133 | 0.14 | 0.889 |
| Study design | -0.1464 | 0.0984 | -1.49 | 0.137 |
| Exposure assessment location | -0.1455 | 0.0822 | -1.77 | 0.077 |
| Exposure window | 0.0166 | 0.0709 | 0.23 | 0.814 |
| Diagnostic method | -0.1539 | 0.0674 | -2.28 | 0.022 |

Table S5. Subgroup analyses of heterogeneity in the association between environmental exposures and ADHD

| **Exposure** | **Factor** | **Category** | **k** | **Pooled estimate**  **(*95% CI*)** | ***I²*(%)** |
| --- | --- | --- | --- | --- | --- |
| Noise | Overall | Overall | 14 | 1.034 (1.014-1.055) | 89 |
| Noise | Region | Europe | 13 | 1.037 (1.016-1.058) | 89.8 |
| Noise | Study design | Cohort | 7 | 1.009 (0.991-1.029) | 56.1 |
| Noise | Study design | Cross-sectional | 7 | 1.053 (1.017-1.089) | 93.9 |
| Noise | Age/exposure window | Postnatal/childhood | 12 | 1.042 (1.018-1.066) | 90.1 |
| Noise | Age/exposure window | Prenatal | 2 | 0.975 (0.934-1.018) | 0 |
| Noise | Exposure location | Home address | 9 | 1.016 (0.998-1.035) | 65.1 |
| Noise | Exposure location | School address | 5 | 1.060 (1.013-1.109) | 95.6 |
| Noise | Diagnostic method | Diagnosis-based | 3 | 1.046 (1.006-1.088) | 58.9 |
| Noise | Diagnostic method | Scale-based | 11 | 1.032 (1.009-1.054) | 90.5 |
| NO_2_ | Overall | Overall | 42 | 1.196 (1.134-1.261) | 98 |
| NO_2_ | Age/exposure window | Postnatal/childhood | 34 | 1.226 (1.150-1.307) | 98.4 |
| NO_2_ | Age/exposure window | Prenatal | 6 | 1.015 (0.976-1.055) | 67.4 |
| NO_2_ | Diagnostic method | Diagnosis-based | 22 | 1.342 (1.255-1.435) | 96 |
| NO_2_ | Diagnostic method | Scale-based | 20 | 1.006 (0.986-1.026) | 71 |
| PM_2.5_ | Overall | Overall | 30 | 1.280 (1.174-1.395) | 96.4 |
| PM_2.5_ | Age/exposure window | Combined prenatal+childhood | 2 | 1.335 (1.335-1.335) | 0 |
| PM_2.5_ | Age/exposure window | Postnatal/childhood | 21 | 1.348 (1.182-1.539) | 97.2 |
| PM_2.5_ | Age/exposure window | Prenatal | 7 | 1.102 (1.038-1.169) | 70.5 |
| PM_2.5_ | Diagnostic method | Diagnosis-based | 15 | 1.347 (1.179-1.538) | 95.3 |
| PM_2.5_ | Diagnostic method | Scale-based | 15 | 1.182 (1.101-1.270) | 89.9 |
| PM_10_ | Overall | Overall | 38 | 1.452 (1.349-1.563) | 96.4 |
| PM_10_ | Age/exposure window | Postnatal/childhood | 28 | 1.765 (1.574-1.978) | 97.3 |
| PM_10_ | Age/exposure window | Prenatal | 8 | 1.011 (0.978-1.046) | 43.8 |
| PM_10_ | Diagnostic method | Diagnosis-based | 24 | 1.919 (1.572-2.343) | 96.7 |
| PM_10_ | Diagnostic method | Scale-based | 14 | 1.041 (1.004-1.079) | 75.4 |
| NOx | Overall | Overall | 12 | 1.025 (0.985-1.066) | 52.7 |
| NOx | Age/exposure window | Prenatal | 11 | 1.026 (0.986-1.068) | 56.7 |
| NOx | Diagnostic method | Diagnosis-based | 7 | 1.085 (1.026-1.147) | 13.7 |
| NOx | Diagnostic method | Scale-based | 5 | 0.985 (0.975-0.995) | 0 |

Fig.S1 Funnel plots of the association between noise exposure and ADHD

Fig.S2 Sensitivity analysis diagram of the association between noise exposure and ADHD

Fig.S3 Funnel plot of the association between NO_2_ exposure and ADHD analyzed using Method A

Fig.S4 Funnel plot of the association between NO_2_ exposure and ADHD analyzed using Method B


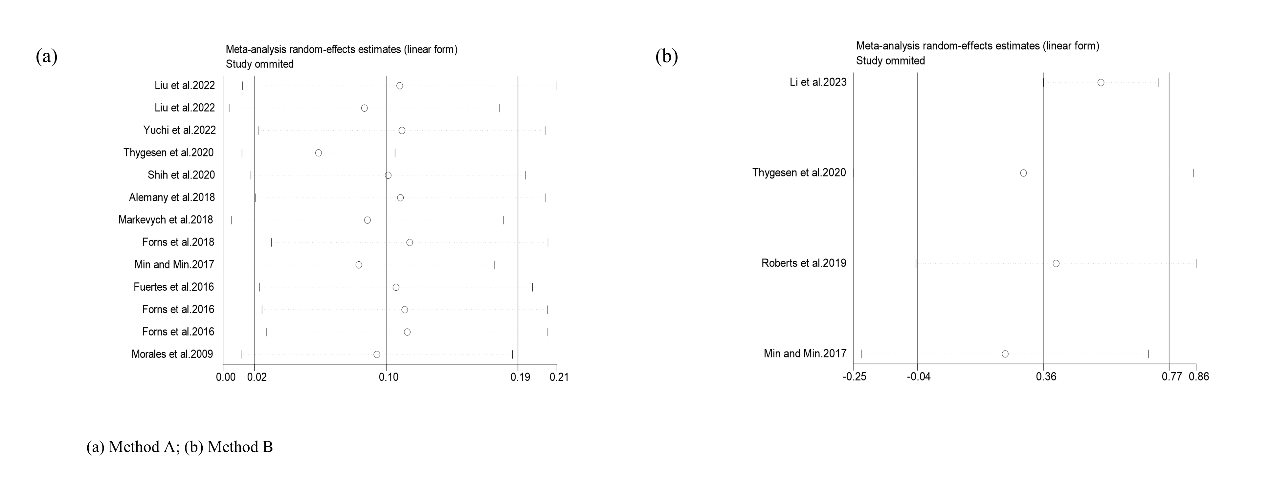
Fig.S5 Sensitivity analysis diagram of the association between NO_2_ exposure and ADHD

Fig.S6 Funnel plot of the association between NOₓ exposure and ADHD

Fig.S7 Sensitivity analysis diagram of the association between NOₓ exposure and ADHD

Fig.S8 Funnel plot of the association between PM_10_ exposure and ADHD analyzed using Method A


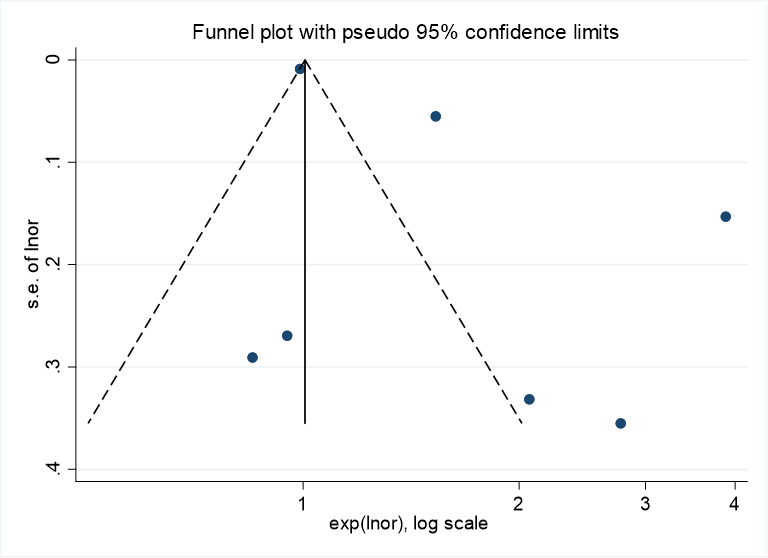


Fig.S9 Funnel plot of the association between PM_10_ exposure and ADHD analyzed using Method B


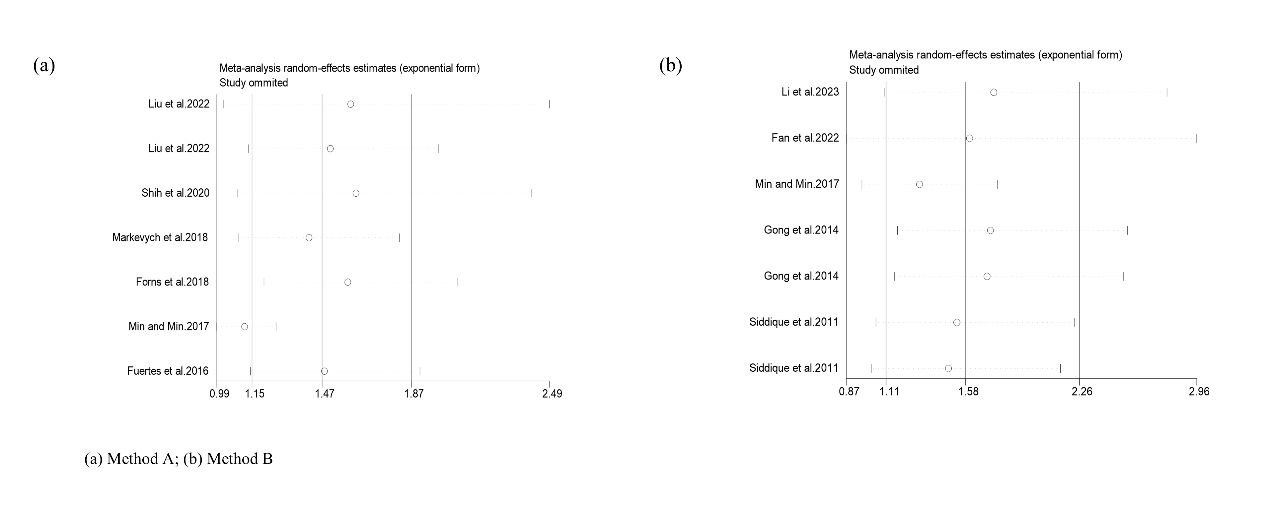


Fig.S10 Sensitivity analysis diagram of the association between PM_10_ exposure and ADHD


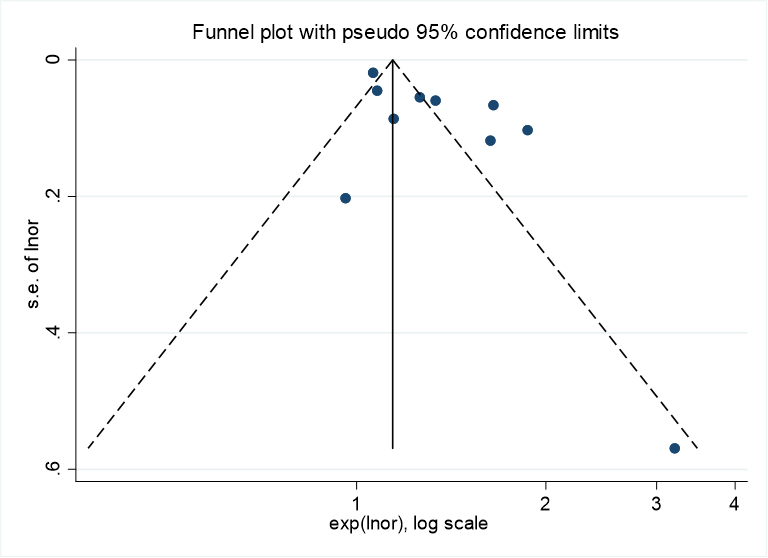


Fig.S11 Funnel plot of the association between PM_2.5_ exposure and ADHD analyzed using Method A

Fig.S12 Funnel plot using the the trim-and-fill method for the association between PM_2.5_ exposure and ADHD analyzed using Method A


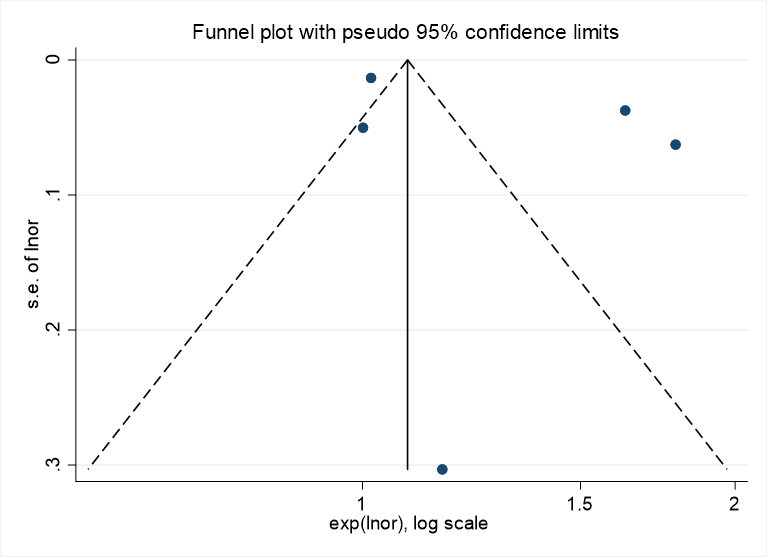
Fig.S13 Funnel plot of the association between PM_2.5_ exposure and ADHD analyzed using Method B


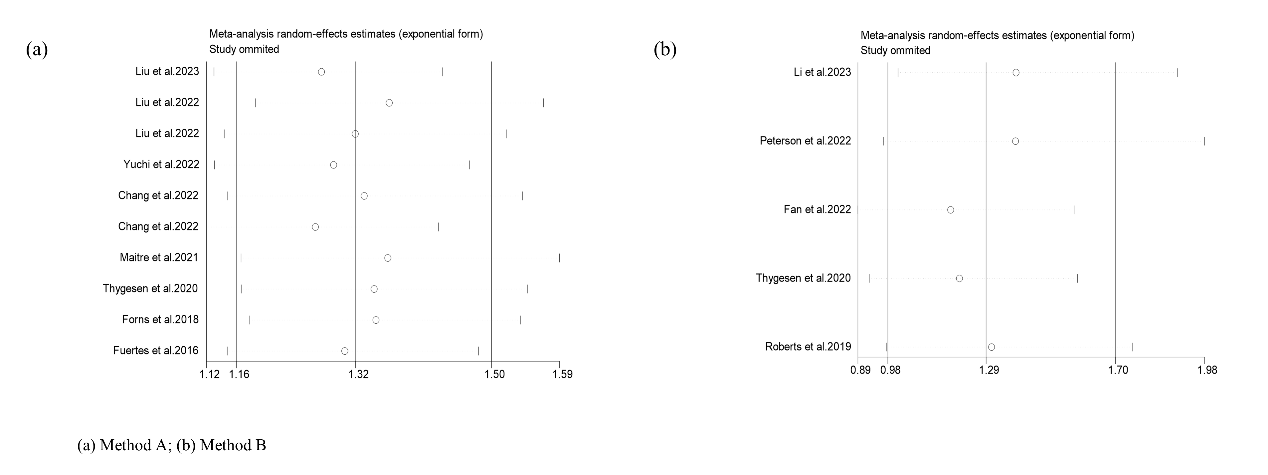


Fig.S14 Sensitivity analysis diagram of the association between PM_2.5_ exposure and ADHD


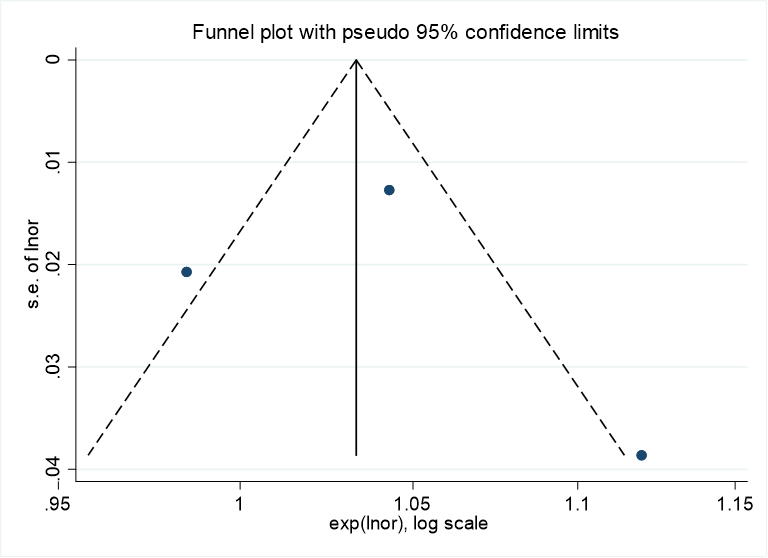
Fig.S15 Funnel plot of the association between O_3_ exposure and ADHD


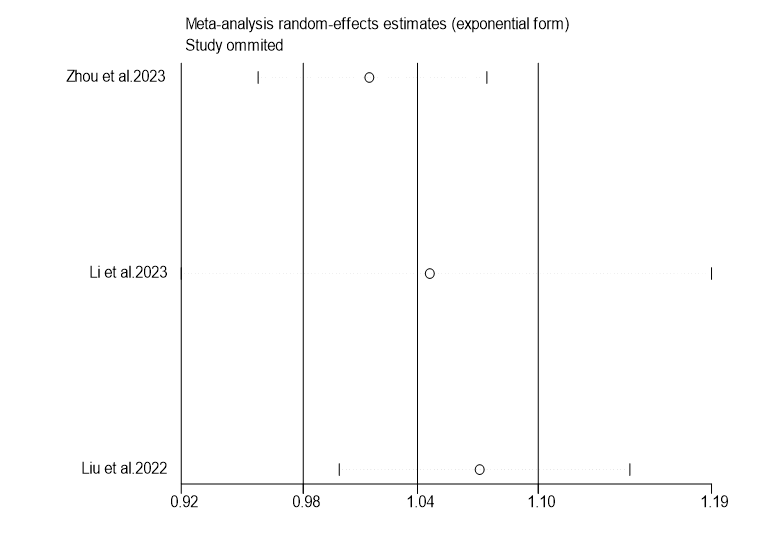


Fig.S16 Sensitivity analysis diagram of the association between O_3_ exposure and ADHD


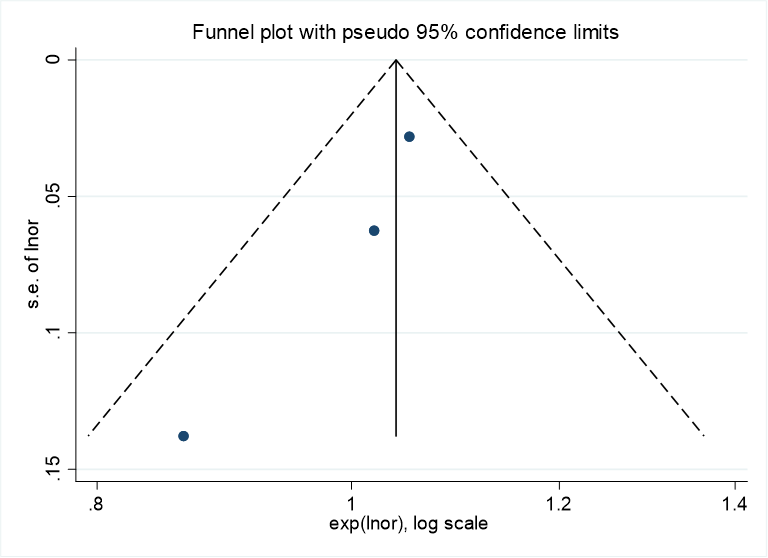
Fig.S17 Funnel plot of the association between SO_2_ exposure and ADHD

Fig.S18 Sensitivity analysis diagram of the association between SO_2_ exposure and ADHD
